# The Dopaminergic Tug of War: A Case of Psychosis at the Crossroads of Endocrinology and Psychiatry During Cabergoline Therapy

**DOI:** 10.1192/bjo.2026.11776

**Published:** 2026-06-30

**Authors:** Kaynat Riaz, Imtiaz Ahmad Dogar

**Affiliations:** Allied II Hospital, Faisalabad Medical Univerity, Faisalabad, Pakistan

## Abstract

**Aims:**

Dopamine agonists like cabergoline are considered first-line therapies for prolactin-secreting pituitary microadenomas. Although efficacious from an endocrinological perspective, the use of dopaminergic stimulation can potentially exacerbate or unmask psychiatric symptoms, including psychosis. The management of emergent psychosis in this scenario is a challenge, as antipsychotic dopamine blockade can potentially worsen hyperprolactinemia. Aripiprazole, a dopamine D_2_ partial agonist, is often preferred for its antipsychotic properties and relative prolactin neutrality; however, its efficacy in the treatment of severe psychosis while on dopamine agonist therapy is unclear.

**Methods:**

A 29-year-old woman with a 12-year history of obsessive-compulsive symptoms, who had remained stable and had never been hospitalized, was diagnosed with hyperprolactinemia after the development of galactorrhea and menstrual disturbances. MRI showed a 6 mm pituitary microadenoma, and cabergoline 0.5 mg twice a week was started with good biochemical control. Shortly thereafter, she experienced severe psychiatric deterioration, including persecutory delusions, ideas of reference, refusal of food, severe self-neglect, and social withdrawal, requiring psychiatric hospitalization. Mental state examination showed prominent psychotic features coexisting with the patient’s previous obsessive phenomena.

Aripiprazole was started at 5 mg/day and gradually increased to 20 mg/day over three weeks, chosen for its partial dopamine agonist properties and prolactin-sparing action. Despite optimal dosing, good tolerability, and maintenance of prolactin levels, there was little relief from psychosis or behavioral disturbance. Because of persistence of severity, aripiprazole was stopped and olanzapine started at titrated upto 15 mg/day. An SSRI was continued for obsessive symptoms. Cabergoline was continued under strict endocrinological follow-up.

**Results:**

Within two weeks of the initiation of olanzapine, there was a marked improvement in persecutory delusions and behavioral disturbance, along with the return of appetite, sleep, and self-care, and partial recovery of insight. Serum prolactin levels remained within acceptable limits (46 ng/mL), and follow-up MRI at three months showed no progression of the tumor.

**Conclusion:**

This particular case illustrates the shortcomings of aripiprazole in dealing with severe psychosis, especially in the setting of dopamine agonist therapy, even in the presence of theoretical endocrine benefits. In situations where endocrine function is substantially affected, psychiatric management may need to focus on effective antipsychotic therapy, with close interdisciplinary attention to minimize endocrine risk.

